# The Role of the Focal Adhesion Protein PINCH1 for the Radiosensitivity of Adhesion and Suspension Cell Cultures

**DOI:** 10.1371/journal.pone.0013056

**Published:** 2010-09-28

**Authors:** Veit Sandfort, Iris Eke, Nils Cordes

**Affiliations:** 1 OncoRay - Center for Radiation Research in Oncology, Medical Faculty Carl Gustav Carus, Dresden University of Technology, Dresden, Germany; 2 Department of Radiation Oncology, University Hospital and Medical Faculty Carl Gustav Carus, Dresden University of Technology, Dresden, Germany; 3 Department of Medicine and Cardiology, Heart Center Dresden University Hospital, Dresden University of Technology, Dresden, Germany; Mizoram University, India

## Abstract

Focal adhesion (FA) signaling mediated by adhesion to extracellular matrix and growth factor receptors contributes to the regulation of the cellular stress response to external stimuli. Critical to focal adhesion assembly and signaling is the adapter protein PINCH1. To evaluate whether the prosurvival function of PINCH1 in radiation cell survival depends on cell adhesion, we examined *PINCH1* ^fl/fl^ and *PINCH1* ^−/−^ mouse embryonic fibroblasts and human cancer cell lines. Here, we found that the enhanced cellular radiosensitivity mediated by PINCH1 depletion observed under adhesion conditions is conserved when cells are irradiated under suspension conditions. This unsuspected finding could not be explained by the observed modification of adhesion and growth factor associated signaling involving FAK, Paxillin, p130^CAS^, Src, AKT, GSK3β and ERK1/2 under suspension and serum withdrawal relative to adhesion conditions with serum. Our data suggest that the adapter protein PINCH1 critically participates in the regulation of the cellular radiosensitivity of normal and malignant cells similarly under adhesion and suspension conditions.

## Introduction

Interactions of cells with the surrounding extracellular matrix (ECM) are inevitable for cell survival [1], [2], [3]. Cell adhesion molecules of the integrin family facilitate these interactions, which are localized to distinct cell membrane areas called focal adhesions (FAs) [2]. Apart from structurally linking ECM, integrins and actin cytoskeleton to ensure tissue integrity and cellular architecture, FAs serve as multiprotein signaling complexes [2], [4], [5]. At these sites, mutual communication between integrin and growth factor receptors required for optimal downstream signaling takes place via the cytoplasmic network to control e.g. proliferation, differentiation, survival and reaction to external stress [6], [7], [8], [9], [10]. For proper FA signaling, FA assembly has to be accomplished by a diverse set of membrane and cytoplasmic proteins including cell adhesion molecules, growth factor receptors as well as adapter and structural proteins such as actin, Rho GTPases and the Particularly Interesting New Cysteine-Histidine-rich proteins (PINCH) 1 and 2 [11], [12].

PINCH1 forms a ternary protein complex with Integrin-Linked Kinase (ILK) and Parvin that essentially contributes to FA assembly and the regulation of cell survival, migration and adhesion processes as shown in avian and mouse models [12], [13], [14], [15], [16]. The expression of these three proteins is mutually interdependent [17]. PINCH1 is a five Lin-11, Isl-1, Mec-3 (LIM) domain-containing adapter protein also interacting with Nck adapter protein 2 [18], Ras suppressor protein 1 (RSU1) [19], [20] and Thymosin β4 [21]. Prosurvival signaling of the PINCH/ILK/Parvin complex involves the Ras/ERK1/2 cascades via RSU1 [19] and the Phosphatidylinositol-3 kinase (PI3K)/Protein kinase B/AKT (AKT) pathway [22]. As compared to Li et al. who did not observe a PINCH1-related AKT phosphorylation [11], recent evidence demonstrated a direct regulatory interaction of PINCH1 with protein phosphatase 1α (PP1α) and AKT1 [23]. Using models of mouse embryonic fibroblasts (MEF) and human cancer cells lines, we were able to show that the PINCH1/PP1α/AKT1 interrelation is responsible for increased cellular resistance to both X-ray irradiation and the chemotherapeutics cisplatin, 5-fluorouracil and gemcitabine [23]. In support of the role of PINCH1 in tumor cell resistance, PINCH1 revealed to be overexpressed in human malignancies relative to normal corresponding tissues [23], a finding confirming the data reported by Wang-Rodriguez et al. [24]. Interestingly, as PINCH1 is located at FAs, the resistance phenotype was not influenced by adhesion to different ECM proteins, which raised the question dealt with in this study whether PINCH1 is able to confer its prosurvival role under lack of adhesion.

Prosurvival signals are transmitted via numerous pathways including AKT, which plays key roles in apoptosis, tumor growth, therapy resistance of tumor cells and radiation responsiveness [25], [26], [27], [28], [29]. Upon phosphorylation at amino acid residues Serine (Ser) 473 and Threonine (Thr) 308, fully activated AKT1 signals to Bad and glycogen synthase kinase 3β (GSK3β) in a PI3K-dependent manner [30]. Inducers of AKT kinase activity are transmembrane growth factor receptors and integrins [27], [31], [32].

Another pathway of integrin-mediated signaling recruits the non-receptor bound 125 kDa protein kinase Focal Adhesion Kinase (FAK) [33]. The phosphorylation of FAK at the autophosphorylation site Tyrosine (Tyr) 397 is a strong indicator of cell adhesion resulting from FAK recruitment to cytoplasmic integrin tails [34]. Subsequently, Src family members phosphorylate additional amino acid residues like Tyr^576^ and Tyr^577^ for full FAK activation [35]. Activated, Tyr^416^ phosphorylated Src induces phosphorylation of p130^CAS^ at Tyr^410^ and Paxillin at Tyr^31^ for mediating prosurvival and proliferative biochemical cues [35]. In addition to FAK overexpression in human tumors detected by immunohistochemistry [36], [37], [38], studies in mouse fibroblasts and various human cancer cell lines from different origin like lung and pancreas suggested that FAK plays a crucial role in the cellular stress response to ionizing radiation [39], [40]. It was shown that FAK overexpression confers radioresistance in leukemia cells while FAK knockdown sensitizes cells from solid tumors to X-ray irradiation [40], [41].

To evaluate whether the prosurvival function of PINCH1 in radiation cell survival depends on cell adhesion or is also conserved in suspension, we examined the clonogenic survival and expression and phosphorylation of a selected panel of prosurvival proteins in *PINCH1* ^fl/fl^ and *PINCH1* ^−/−^ mouse embryonic fibroblasts and human cancer cell lines grown either adherently or in suspension.

## Materials and Methods

### Antibodies and reagents

Antibodies against ILK, FAK, GSK3β (BD, Heidelberg, Germany), α-Parvin (Acris, Hiddenhausen, Germany), FAK Tyr397 (Calbiochem, Darmstadt, Germany), AKT, AKT Ser473, FAK Tyr576/577, GSK3β Ser9, p130^CAS^ Tyr410, Src Tyr416, ERK1/2, ERK1/2 Thr202/Tyr204 (Cell Signaling, Frankfurt, Germany), Paxillin, Paxillin Tyr31, β-Actin (Sigma, Taufkirchen, Germany), p130^CAS^, phospho-Tyrosine, Src, phospho-Histon H2AX-S139 (Upstate, Lake Placid, USA), PINCH1/2 (Santa Cruz, Heidelberg, Germany), 53BP1 (Novus, Littleton, USA), Alexa594 anti-mouse, Alexa488 anti-rabbit Alexa594 phalloidin (Invitrogen, Karlsruhe, Germany) and horseradish peroxidase-conjugated donkey anti-rabbit and sheep anti-mouse (Amersham, Freiburg, Germany) antibodies were purchased as indicated. Antibody against PINCH1 was a generous gift from Prof. Fässler (Max-Planck Institute of Biochemistry, Martinsried, Germany). Enhanced chemiluminescent reagent (ECL) was from Amersham (Freiburg, Germany) and Vectashield/DAPI mounting medium from Alexis (Grünberg, Germany).

### Cell culture and radiation exposure

*PINCH1* ^fl/fl^, *PINCH1* ^−/−^, EGFP-PINCH1, *ILK* ^fl/fl^ and *ILK* ^−/−^ cells were kindly provided by Prof. Fässler (Max-Planck Institute of Biochemistry, Martinsried, Germany). Tumor cell lines A172, U138MG, A549, SKMES1, CCL221, HTB35, HTB43, MDAMB231, PATU8902, Jurkat, HL60 were obtained from the American Type Culture Collection (Manassas, VA, USA). CCD32 and HSF1/2 were a generous gift from Prof. Rodemann (University Tübingen, Germany). *PINCH1* ^fl/fl^ and *PINCH1* ^−/−^ MEFs and EGFP and EGFP-tagged full-length PINCH1 (EGFP-PINCH1) expressing PINCH1-deficient fibroblasts were generated as previously described [12]. Cells were cultured in Dulbecco's Modified Eagle Medium (DMEM), MEM (U138MG) or RPMI (CCL221, Jurkat, HL60) containing Glutamax-I supplemented with 10% fetal calf serum and 1% non-essential amino acids (PAA, Cölbe, Germany) at 37°C in a humidified atmosphere containing 10% CO_2_ or 5% CO_2_ (CLL221, Jurkat, HL60), pH 7.4. In all experiments, asynchronously growing cells were used. Irradiation was delivered at room temperature using single doses of 200 kV X-rays (Yxlon Y.TU 320; Yxlon, Copenhagen, Denmark) filtered with 0.5 mm Cu. The absorbed dose was measured using a Duplex dosimeter (PTW, Freiburg, Germany). The dose-rate was approximately 1.3 Gy/min at 20 mA and applied doses ranged from 0 to 6 Gy.

### Suspension cultures

For suspension cultures, plates were coated with 1% agarose to prevent cell attachment. Cells were trypsinized, washed with PBS and kept in suspension with or without FCS for indicated time intervals at 37°C in a humidified atmosphere containing 10% CO_2_.

### Colony formation assay

The colony formation assay was applied for measurement of clonogenic cell survival as published [42]. Cells were grown on uncoated (Poly-S, polystyrene) culture dishes or dishes precoated with Fibronectin (FN; BD, Heidelberg, Germany) or poly-L-Lysine (Poly-L; Sigma, Taufkirchen, Germany) (all at 1 µg/cm^2^). Cells were irradiated 24 or 48 hours after plating. For irradiation in suspension, cells were detached, washed with 1XPBS and cultured in suspension with or without serum for 1 h as described above. Then, suspension cell cultures were irradiated and plated onto FN, Poly-L or Poly-S at indicated time intervals. Cell colonies with a minimum of 50 cells were microscopically counted at 8 days (*PINCH1* ^fl/fl^, *PINCH1* ^−/−^), 11 days (HTB43) or 14 days (HTB35) after plating. Plating efficiencies were calculated as follows: numbers of colonies formed/numbers of cells plated. Surviving fractions (SF) were calculated as follows: numbers of colonies formed/(numbers of cells plated (irradiated)×plating efficiency (unirradiated)). Each point on survival curves represents the mean surviving fraction from at least three independent experiments.

### Proliferation assay

4×10^4^ *PINCH1* ^fl/fl^ or *PINCH1* ^−/−^ cells were plated on FN or Poly-L precoated culture flasks. Cell counting was performed 8 days later by automatic cell counting (Z2 Particle Analyzer, Beckman Coulter, Krefeld, Germany). Three independent experiments were performed.

### Analysis of cell number per colony

Determination of cell number per colony size under tested conditions was performed to control for putative differences in adhesive capacity, which might impact on measurement of clonogenic cell survival. After 8 days (*PINCH1* ^fl/fl^, *PINCH1* ^−/−^), 11 days (HTB43) or 14 days (HTB35), cells were fixed and stained with Coomassie and cell numbers of 15 colonies were counted microscopically.

### Total protein extracts and Western Blotting

Adherent cells were rinsed with ice-cold 1XPBS prior to harvesting total proteins by scraping using modified RIPA buffer (50 mM Tris-HCl (pH 7.4), 1% Nonidet-P40, 0.25% sodium deoxycholate, 150 mM NaCl, 1 mM EDTA, Complete protease inhibitor cocktail (Roche, Mannheim, Germany), 1 mM Na_3_VO_4_, 2 mM NaF). Cells in suspension were centrifuged and washed with ice-cold 1XPBS. The supernatant was discarded and the remainder was lysed with modified RIPA buffer. Samples were stored at −80°C. Total protein amounts were measured with the BCA assay (Pierce, Bonn, Germany). After SDS-PAGE and transfer of proteins onto nitrocellulose membranes (Schleicher and Schuell, Dassel, Germany), probing and detection of specific proteins was accomplished with indicated antibodies and ECL as described [42].

### siRNA transfection

PINCH1 siRNA (sequence: 5′-GGACCUAUAUGAAUGGUUUtt-3′) was obtained from Applied Biosystems (Darmstadt, Germany) and the non-specific control siRNA (sequence: 5′-GCAGCUAUAUGAAUGUUGUtt-3′ from MWG (Ebersberg, Germany). siRNA delivery was accomplished as published [23]. Twenty-four hours after transfection with oligofectamine (Invitrogen, Darmstadt, Germany) and 20 nM siRNA under serum-free conditions, cells were plated and irradiated after 24 h or irradiated after 1 h in suspension. Colony formation assays were performed. Efficient PINCH1 knockdown was confirmed by Western blotting 48 h after transfection.

### Adhesion assay

Modification of cell adhesion upon PINCH1 depletion was scored using an adhesion assay. HTB43 and HTB35 cells were transfected with non-specific control siRNA or PINCH1 siRNA. After 48 h, cells were kept in suspension for 1 h before plating on culture plastic. Fixation with 70% ethanol and staining with Coomassie was performed at indicated time points upon removal of non-attached cells using 1XPBS. Four defined fields per 35 mm-well were microscopically (Axiovert 25, Zeiss) evaluated for the number of adherent cells.

### Immunofluorescence staining

To provide further mechanistic insight into the enhanced radiosensitivity after PINCH1 silencing, we measured residual DNA-double strand breaks (rDSB) by using the foci assay. As previously published [42], [43], rDSBs were visualized by double staining of phosphorylated H2AX (γH2AX) plus p53 binding protein-1 (53BP1). PINCH1 HTB43 and HTB35 knockdown cell cultures were fixed with 1% formaldehyde/PBS at 24 h after X-ray irradiation (0 or 6 Gy). Permeabilization with 0.25% Triton X-100/PBS preceded staining with specific anti-γH2AX and anti-53BP1 antibodies and Vectashield/DAPI mounting medium. γH2AX/53BP1-positive nuclear foci of at least 150 cells from three independent experiments were counted microscopically with an Axioscope 2plus fluorescence microscope (Zeiss) and defined as rDSBs.

### DAPI staining for apoptosis analysis

Knockdown cell cultures were irradiated with 0 and 6 Gy. After 24 h, cells were fixed with 80% ethanol and stained with Vectashield/DAPI mounting medium. At least 100 cells were counted from three independent experiments.

### Data analysis

Means±SD of at least three independent experiments were calculated with reference to untreated controls defined in a 1.0 scale. To test statistical significance, Student's t test was performed using Microsoft® Excel 2003. Results were considered statistically significant if a *P*-value of less than 0.05 was reached. Densitometry of Western blots was performed by scanning of the exposed film and using ImageJ analysis software (http://rsbweb.nih.gov/ij/).

## Results

### PINCH1 regulates radiation survival in a substratum-independent manner

**Figure 1:**
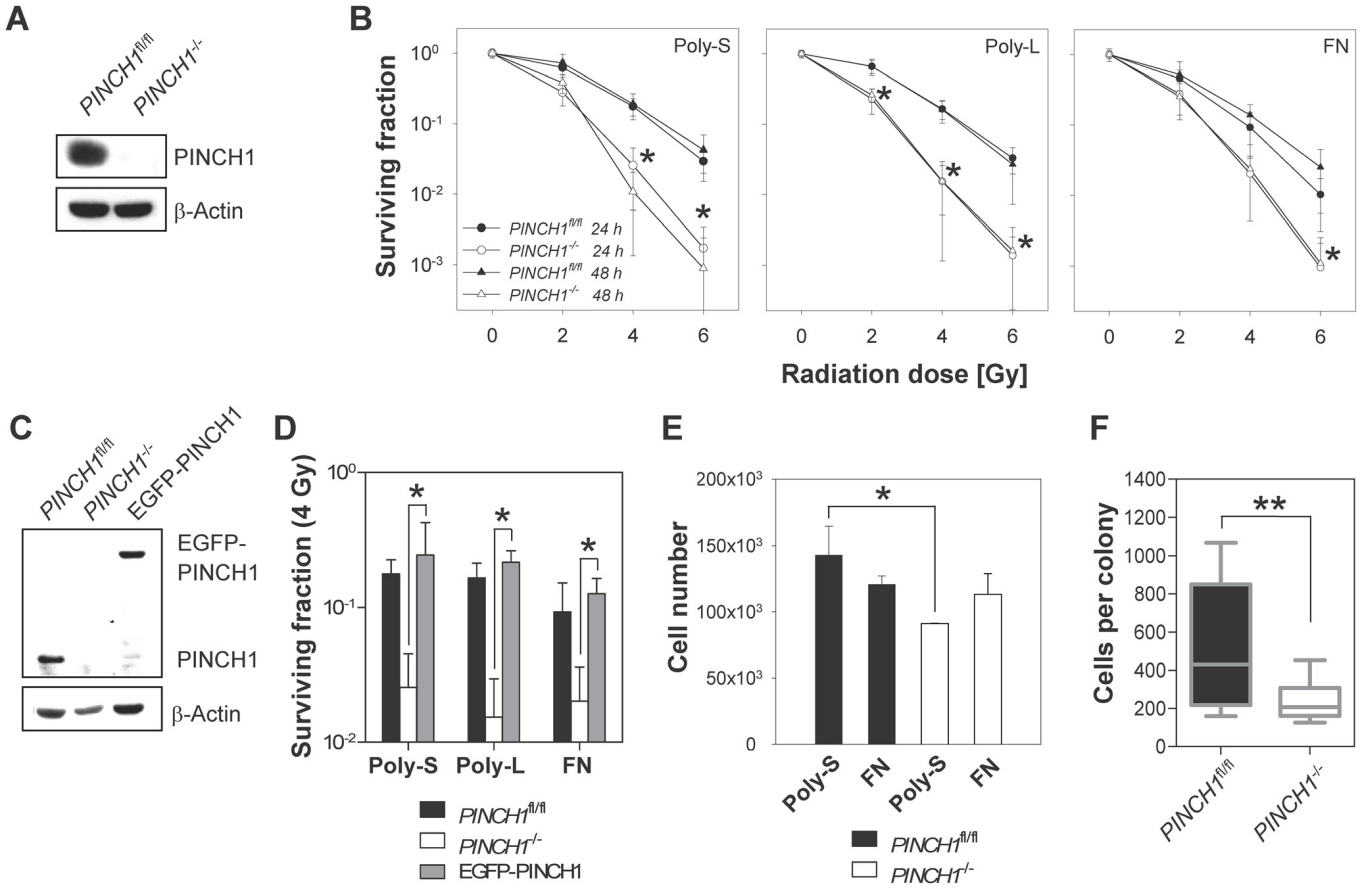
PINCH1 determines radiation cell survival in a substratum-independent manner. (A) Western blot on *PINCH1* ^fl/fl^ and *PINCH1* ^−/−^ MEF lysates. (B) Measurement of clonogenic survival in MEFs irradiated (0–6 Gy X-ray) at 24h or 48h after plating (mean±SD; *n* = 3; * *P*<0.05; t-test). (C and D) Clonogenic survival in non-irradiated and irradiated (4 Gy) *PINCH1* ^fl/fl^ and *PINCH1* ^−/−^ and *PINCH1* ^−/−^ reconstituted with EGFP-PINCH1 MEFs (mean±SD; *n* = 3; t-test). (E) Total cell numbers from colony formation assays at the time point of assay termination ( = day 8). (F) Cell numbers of 15 poly-S colonies were counted. Results are mean±SD (*n* = 3; t-test). Polystyrene, Poly-S; poly-L-Lysine, Poly-L; Fibronectin, FN.

In this study, cells were irradiated on different substrata, i.e. polystyrene (Poly-S), poly-L-lysine (Poly-L) and Fibronectin (FN) at different time periods after plating. In line with our previous observations [23], *PINCH1* ^−/−^ MEF grown on Poly-S, Poly-L and FN were significantly (*P*<0.05) more sensitive to ionizing radiation than *PINCH1* ^fl/fl^ MEF grown on Poly-S, Poly-L and FN (Fig. 1A and B). Interestingly, this was also true when comparing MEFs irradiated with increasing single doses of X-ray 24 h after plating with MEFs irradiated 48 h after plating (Fig. 1B). Thus, cell-to-cell contact (or 2 to 4 cell microcolonies) as observable at the 48-h irradiation schedule seemed dispensable in our hands with regard to colony formation. On top of this, we found similar, substratum-independent radiation survival of *PINCH1* ^fl/fl^ and PINCH1 reconstituted *PINCH1* ^−/−^ cells as compared to *PINCH1* ^−/−^ MEF (Fig. 1C and D). In parallel, measurement of cell numbers at day 8 ( = day of termination of colony formation assay) revealed a significant (*P*<0.05) reduction in the number of *PINCH1* ^−/−^ MEF grown on Poly-S as compared to *PINCH1* ^fl/fl^ MEF, which was not detectable on FN (Fig. 1E). This latter observation, indicating a proliferation difference between *PINCH1* ^−/−^ and *PINCH1* ^fl/fl^ MEFs likely to influence clonogenic survival, was further analyzed by counting the cell numbers of grown colonies. Despite the significant discrepancy in the number of cells per colony of *PINCH1* ^−/−^ relative to PINCH1-expressing MEFs, all representative colonies counted consisted of more than 50 cells as relevant parameter for colony formation assays (Fig. 1F). Additionally, and also with regard to our clonogenic survival analysis, the plating efficiencies of the two MEF cell lines clearly show no survival advantage for *PINCH1* ^fl/fl^ over *PINCH1* ^−/−^ cells (Table 1).

**Table 1:** Plating efficiencies of *PINCH1* ^fl/fl^ and *PINCH1* ^−/−^ MEFs under indicated growth conditions.

Condition	Plating efficiency	
	*PINCH1* ^fl/fl^	*PINCH1* ^−/−^
**Poly-S** * **(24 h)**	0.104+/−0.012	0.143+/−0.078
**Poly-L (24 h)**	0.088+/−0.010	0.156+/−0.046
**FN (24 h)**	0.082+/−0.019	0.093+/−0.025
**Suspension (Poly-S)**	0.084+/−0.034	0.199+/−0.055
**Suspension (Poly-L)**	0.056+/−0.005	0.172+/−0.100
**Suspension (FN)**	0.049+/−0.010	0.088+/−0.008

*Poly-S, polystyrene; Poly-L, poly-L-lysine; FN, Fibronectin.

### PINCH1 knockout radiosensitivity is conserved under suspension conditions

**Figure 2:**
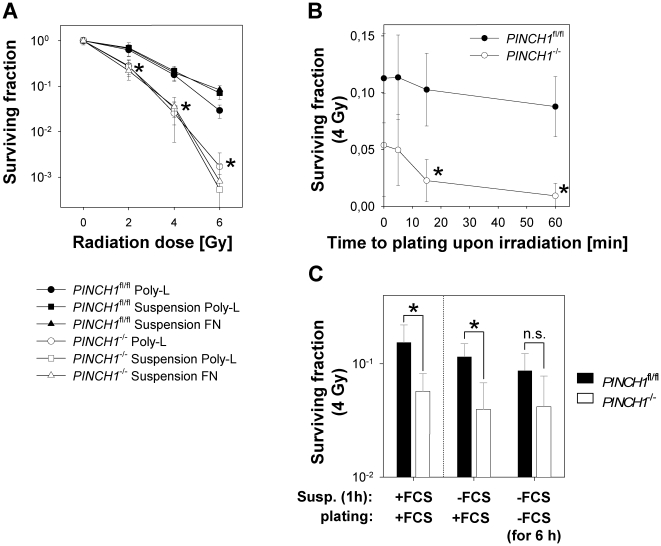
Adhesion and suspension preconditioning mediates similar radiation cell survival of *PINCH1* ^fl/fl^ and *PINCH1* ^−/−^ MEF. (A) Clonogenic survival of *PINCH1* ^fl/fl^ and *PINCH1* ^−/−^ MEFs irradiated under adhesion to Poly-L or after 1h in suspension with 0–6 Gy X-rays. After irradiation, suspension cell cultures were plated on Poly-L or FN for colony formation (mean±SD; *n* = 3; t-test). (B) Colony formation was determined in cells held in suspension for 1h prior to a 4-Gy X-ray irradiation. Then, cells were plated on FN at indicated time point (mean±SD; *n* = 3; t-test). (C) Cells were kept in suspension for 1h with (+FCS) or without FCS (-FCS) prior to 4-Gy irradiation. Subsequently, cell plating to FN was performed under two conditions: +FCS = plating with FCS; -FCS (for 6 h) = FCS was added 6 h after plating. Poly-L-Lysine, Poly-L; Fibronectin, FN; FCS, fetal calf serum. * *P*<0.05. ns, not significant.

To further assess the substratum independency of PINCH1 knockout-mediated radiosensitization, we kept cells in suspension for 1 h prior to irradiation and re-plating. Strikingly, clonogenic radiation survival of suspension cultures was superimposable to that of adhesion Poly-L cultures including retained radiosensitization by PINCH1 knockout (Fig. 2A). Moreover, *PINCH1* ^−/−^ cell cultures kept in suspension for additional 15 or 60 min after 4-Gy irradiation indicated a conserved radiosensitization throughout the observed time period as compared to *PINCH1* ^fl/fl^ MEF (Fig. 2B). Comparing these data generated under suspension plus FCS with data generated under suspension minus FCS revealed that *PINCH1* ^−/−^ MEF maintain their level of radiation cell survival independent from FCS over a time interval of 7 h while the radiation survival of *PINCH1* ^fl/fl^ MEF showed a reduction with prolonged FCS withdrawal (Fig. 2C). Conclusively, these data strongly indicate that PINCH1 is a prosurvival factor independent from the cell's adhesive status in cells exposed to ionizing radiation.

### Signal transduction by PINCH1 under adhesion and suspension

**Figure 3:**
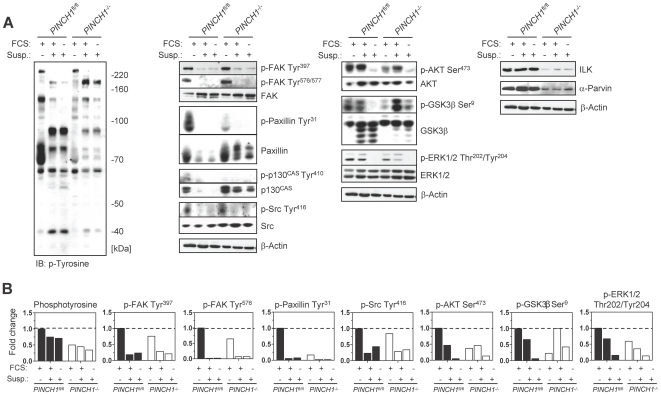
Effects of PINCH1 knockout on signal transduction under adhesion and suspension conditions. (A) Western blot on total cell lysates from *PINCH1* ^fl/fl^ and *PINCH1* ^−/−^ MEF grown on polystyrene plus FCS (first lane), in suspension plus FCS (second lane) or in suspension without FCS (third lane). (B) Densitometric analysis from protein bands shown in ‘A’ after normalization to total protein or β-Actin expression and subsequently to adhesion plus FCS conditions of *PINCH1* ^fl/fl^ MEF ( = 1). FCS, fetal calf serum; Susp, suspension.

To gain insights into PINCH1 signaling, which has been connected to both integrin and growth factor receptor pathways [4], [18], we next sought to examine protein expression and phosphorylation under adhesion and suspension conditions similar to those accomplished for clonogenic survival. Comparable to reduced phosphotyrosine levels in adherent *PINCH1* ^−/−^ MEF (Fig. 3A and B), phosphorylation of FAK Tyr^397^ and Tyr^576/577^, Paxillin Tyr^31^, p130^CAS^ Tyr^410^, Src Tyr^416^, AKT Ser^473^, GSK3β Ser^9^ and ERK1/2 Thr^202^/Tyr^204^ was diminished by up to 80% relative to *PINCH1* ^fl/fl^ MEF (Fig. 3A and B). Despite a lack of further strong tyrosine dephosphorylation under suspension conditions in the presence or absence of serum, FAK, Paxillin, Src and ERK1/2 indicated an additional decrease in their specific phosphorylations in *PINCH1* ^fl/fl^ and *PINCH1* ^−/−^ suspension cultures (Fig. 3A and B). Phosphorylated AKT Ser^473^ was only further reduced under suspension plus serum starvation (Fig. 3A and B). Interestingly, except for phosphotyrosine, the phosphorylation levels of *PINCH1* ^fl/fl^ and *PINCH1* ^−/−^ MEFs showed great similarity when non-adherent and serum starved (Fig. 3A and B). Concerning total protein expression, ILK and α-Parvin, two PINCH1 interacting proteins, were repressed, AKT, GSK3β and ERK1/2 stayed unaltered and FAK and its associated proteins p130^CAS^, Paxillin and Src were slightly induced (FAK, Src) or reduced (p130^CAS^, Paxillin) in case of suspension and serum absence (Fig. 3A). Intriguingly, alterations of p130^CAS^, Paxillin and Src were more prominent in *PINCH1* ^fl/fl^ than in *PINCH1* ^−/−^ MEF (Fig. 3A).

### Expression of the complex components ILK, PINCH1 and α-Parvin in human tumor and mouse cell lines

**Figure 4:**
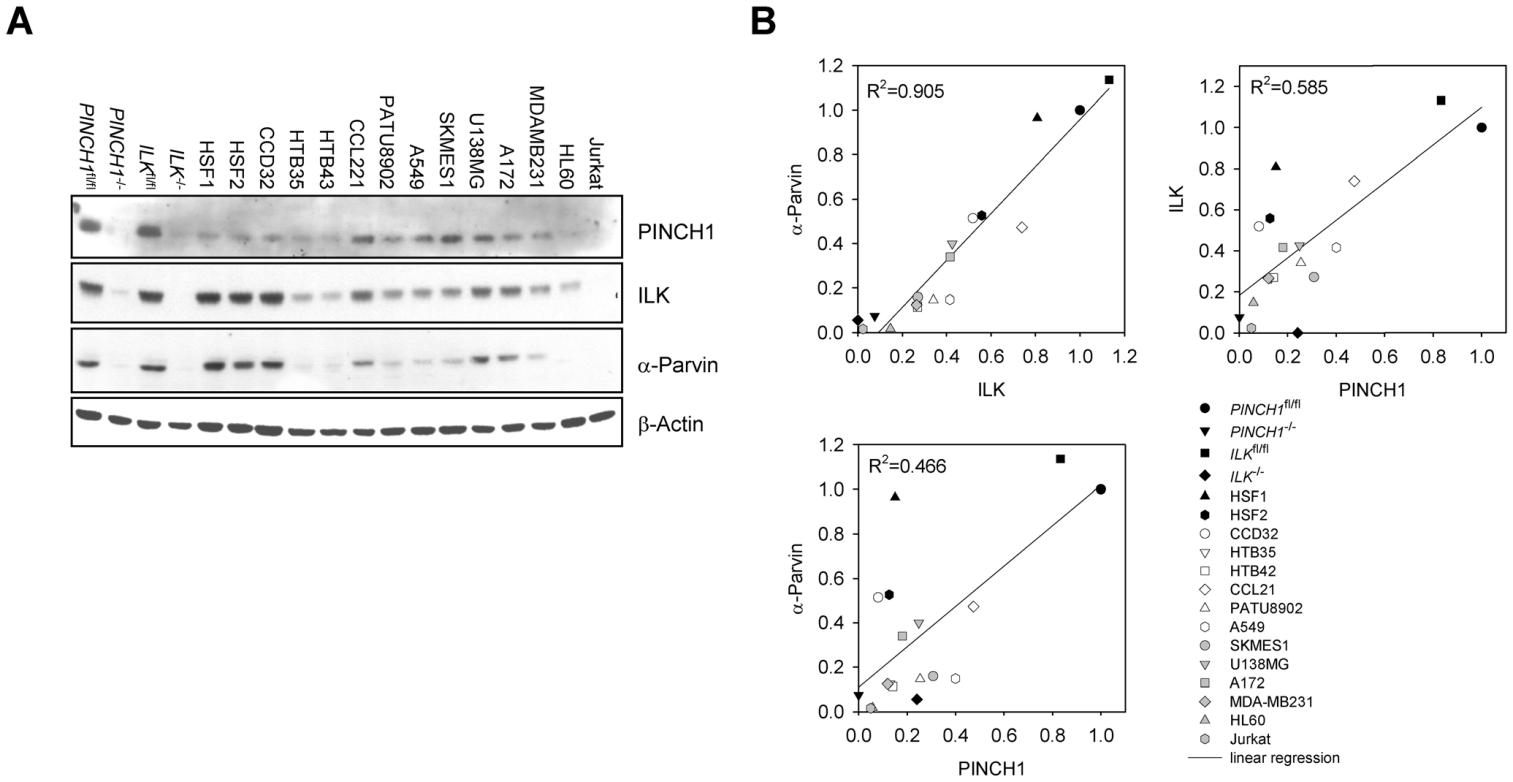
Characterization of PINCH1, ILK and α-Parvin expression in different human tumor and immortalized mouse cell lines. (A) Western blot on total cell lysates of human and mouse cell lines from different origin (mouse = *PINCH1* ^fl/fl^ MEF, *PINCH1* ^−/−^ MEF, *ILK* ^fl/fl^ renal fibroblasts, *ILK* ^−/−^ renal fibroblasts; human primary fibroblasts = HSF1, HSF2, CCD32; human cancers = HTB35 (cervix), HTB43 (pharynx), CCL221 (colon), PATU8902 (pancreas), A549, SKMES1 (lung), U138MG, A172 (brain), MDAMB231 (breast), HL60, Jurkat (leukemia). (B) Correlation between the protein expressions of ILK versus PINCH1 versus Parvin in the different cell lines as analyzed in ‘A’.

From cell biology experiments, we know that PINCH1, ILK and α-Parvin are directly interacting proteins involved in focal adhesion assembly and that their expression is mutually dependent [14], [17]. In accordance with their important function in prosurvival signaling, we characterized the expression of these three proteins in normal mouse and human normal fibroblasts and human tumor cell lines (Fig. 4A) and calculated their correlation. In line with published observations were the expression patterns of *PINCH1* ^fl/fl^ versus *PINCH1* ^−/−^ and *ILK* ^fl/fl^ versus *ILK* ^−/−^ mouse fibroblasts (Fig. 4A and B). Overall, only the expression of ILK and α-Parvin strongly correlated with a R^2^ of 0.905 (Fig. 4B). The correlations between ILK and PINCH1 (R^2^ = 0.585) and α-Parvin and PINCH1 (R^2^ = 0.466) were poor throughout the examined cell line panel (Fig. 4B). To note, Jurkat leukemia cells lacked expression of all three proteins (Fig. 4A). These data reveal that the expression of PINCH1, ILK and α-Parvin is per se not strongly correlative but rather cell line-dependent.

### PINCH1 depletion enhances tumor cell radiosensitivity under adhesion and suspension conditions

**Figure 5:**
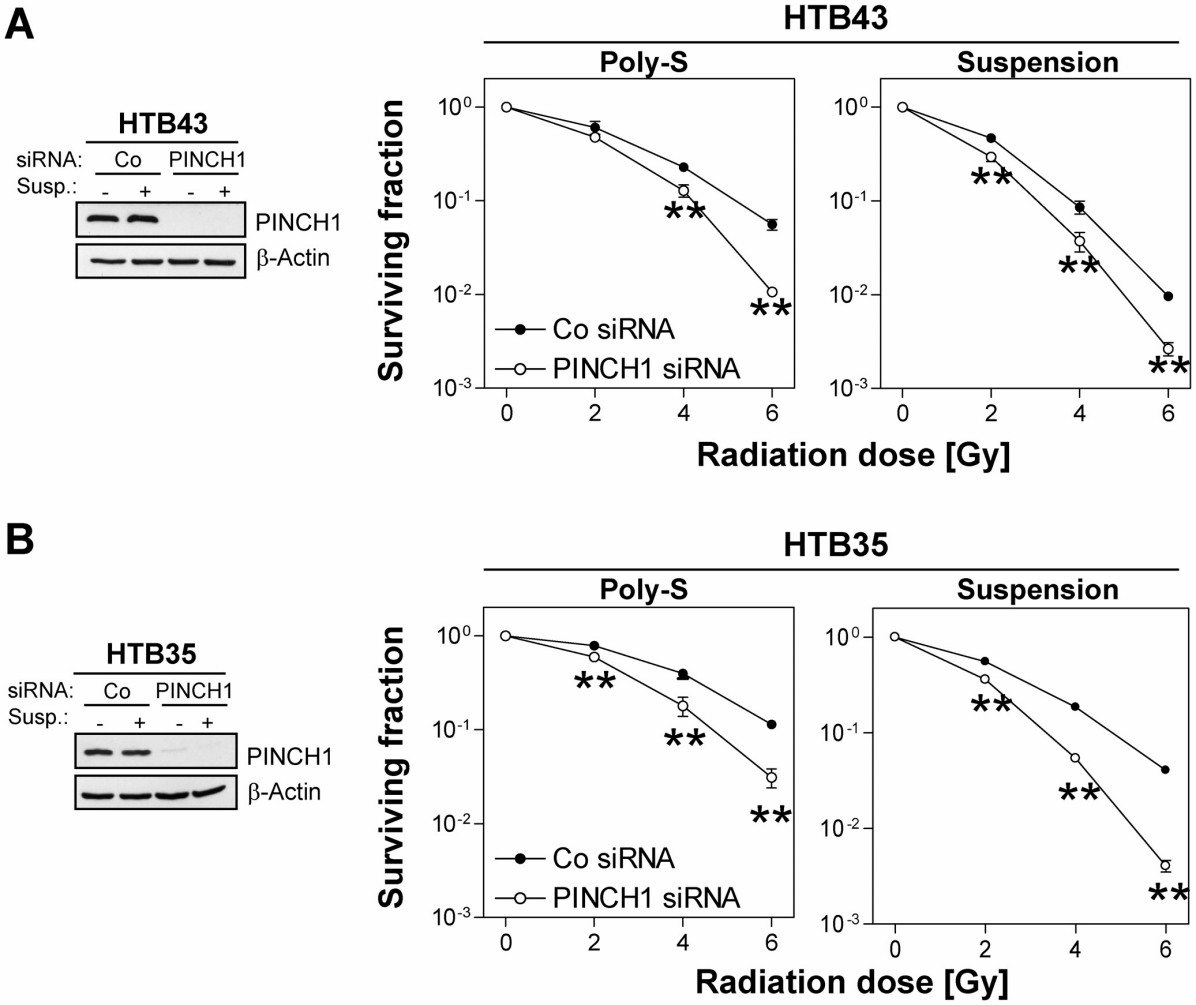
PINCH1 depletion sensitizes human tumor cells to irradiation independent from adhesion. siRNA-mediated PINCH1 depletion was confirmed by Western blotting in human HNSCC cell lines HTB43 (A) and HTB35 (B) cells. Measurement of clonogenic radiation survival (0–6 Gy X-ray) upon PINCH1 knockdown is shown in adherent and suspension (1 h) cultures (mean±SD; *n* = 3; * *P*<0.05, ** *P*<0.01; t-test). Poly-S, Polystyrene; Co, control.

As the presented findings may be of interest for cancer biology and treatment, we assessed the effects of a PINCH1 depletion in adherent and suspension cell cultures of a head and neck squamous cell carcinoma (HTB43) and a cervix carcinoma (HTB35) cell line. While plating efficiencies were not significantly affected by PINCH1 silencing (Table 2), the PINCH1 knockdown resulted in a significantly (*P*<0.01) enhanced radiosensitivity of Poly-S grown HTB43 (Fig. 5A) and HTB35 (Fig. 5B) cells relative to siRNA controls. Intriguingly, suspension conditions mediated a higher degree of radiosensitivity with a conserved (partly stronger) radiosensitization by PINCH1 silencing (Fig. 5A and B). The resulting suspension dose-effect curves of both cell lines were superimposable to the PINCH1 knockdown curves generated in adherent cultures.

**Table 2:** Plating efficiencies of HTB43 and HTB35 under siRNA-mediated PINCH1 knockdown.

Cell line/Condition	Plating efficiency	
	*Co siRNA*	*PINCH1 siRNA*
**HTB43 Poly-S** * **(24 h)**	0.359+/−0.018	0.362+/−0.027
**HTB43 Suspension**	0.520+/−0.035	0.434+/−0.057
**HTB35 Poly-S (24 h)**	0.166+/−0.008	0.207+/−0.019
**HTB35 Suspension**	0.172+/−0.011	0.217+/−0.014

*Poly-S, polystyrene.

**Figure 6:**
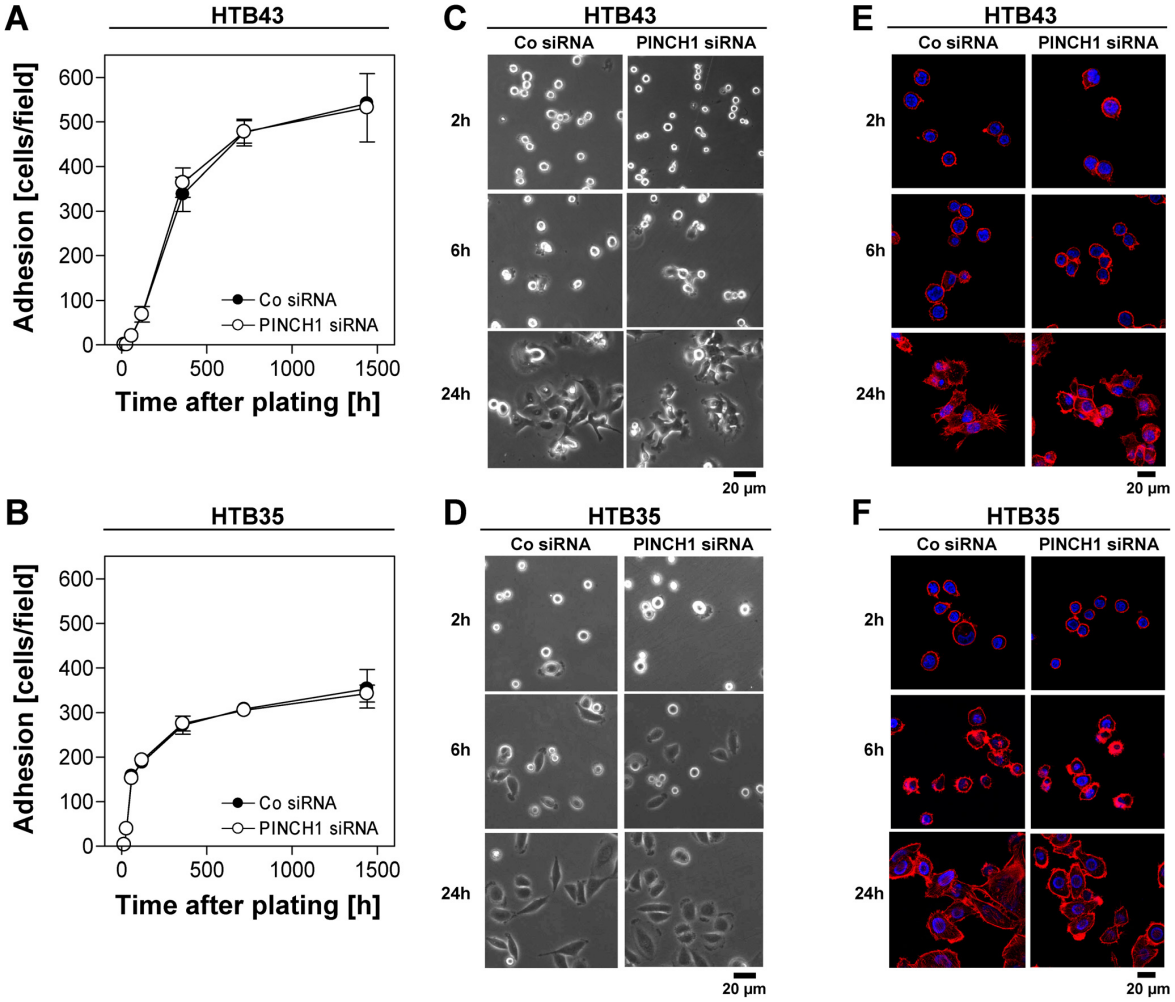
PINCH1 depletion does not modify tumor cell adhesion. Adhesion (cells per field) of (A) HTB43 and (B) HTB35 cells upon PINCH1 knockdown. At 48 h after siRNA transfection, cells were kept in suspension for 1 h on agarose prior to plating. Indicated time points were analyzed for the number of adherent cells as described under “Materials and Methods”. Results show mean±SD (*n* = 3). (C) and (D) display representative microscopic images and (E) and (F) f-actin immunofluorescence stainings (Phalloidin) of HTB43 and HTB35 cells, respectively, at indicated time point after plating.

**Figure 7:**
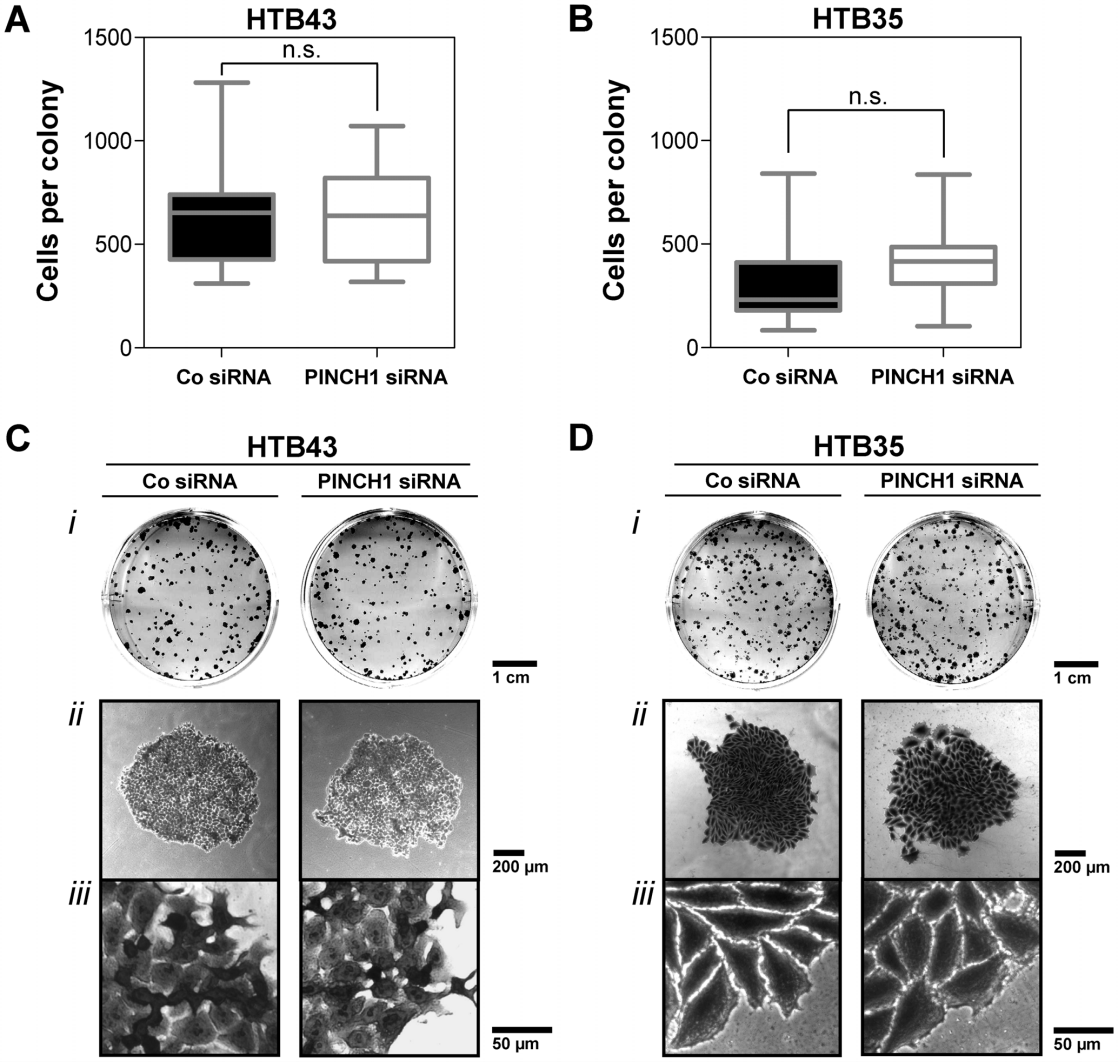
Colony cell numbers and cell morphology remain unaltered upon PINCH1 depletion. (A) and (B) Cell numbers of 15 colonies were counted in PINCH1 knockdown and control HTB43 and HTB35 cell cultures. Results show mean±SD (*n* = 3; t-test; n.s., not significant). (C) and (D) show cellular morphology in PINCH1 depleted and control cell colonies of HTB43 and HTB35 tumor cell lines. Photographs illustrate (*i*) representative colony growth in 35-mm wells, (*ii*) a single representative colony (magnification = 10×), and (*iii*) a representative zoom (magnification = 40×) from the edge of a colony. Co, control.

**Figure 8:**
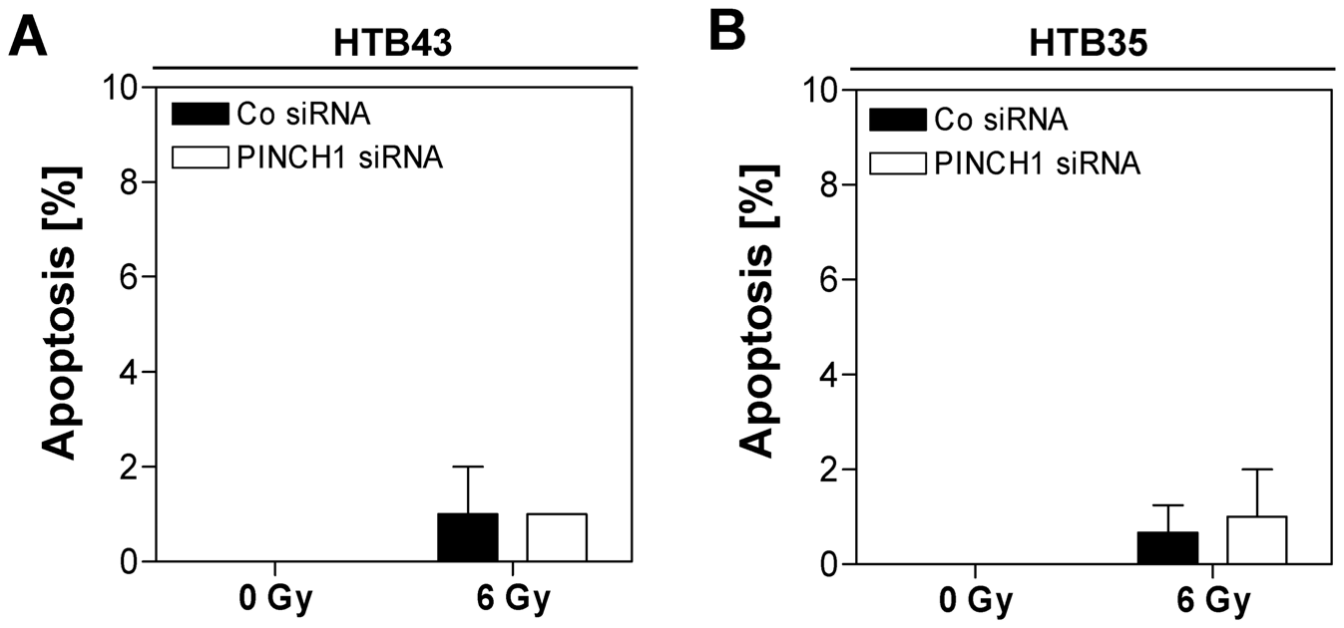
Apoptosis in irradiated cells remains unchanged by PINCH1 silencing. (A) and (B) HTB43 and HTB35 were assessed for typical apoptotic nuclear morphology upon X-ray irradiation (0 or 6 Gy) and transfection with PINCH1 specific or non-specific control siRNA (mean±SD; *n* = 3).

**Figure 9:**
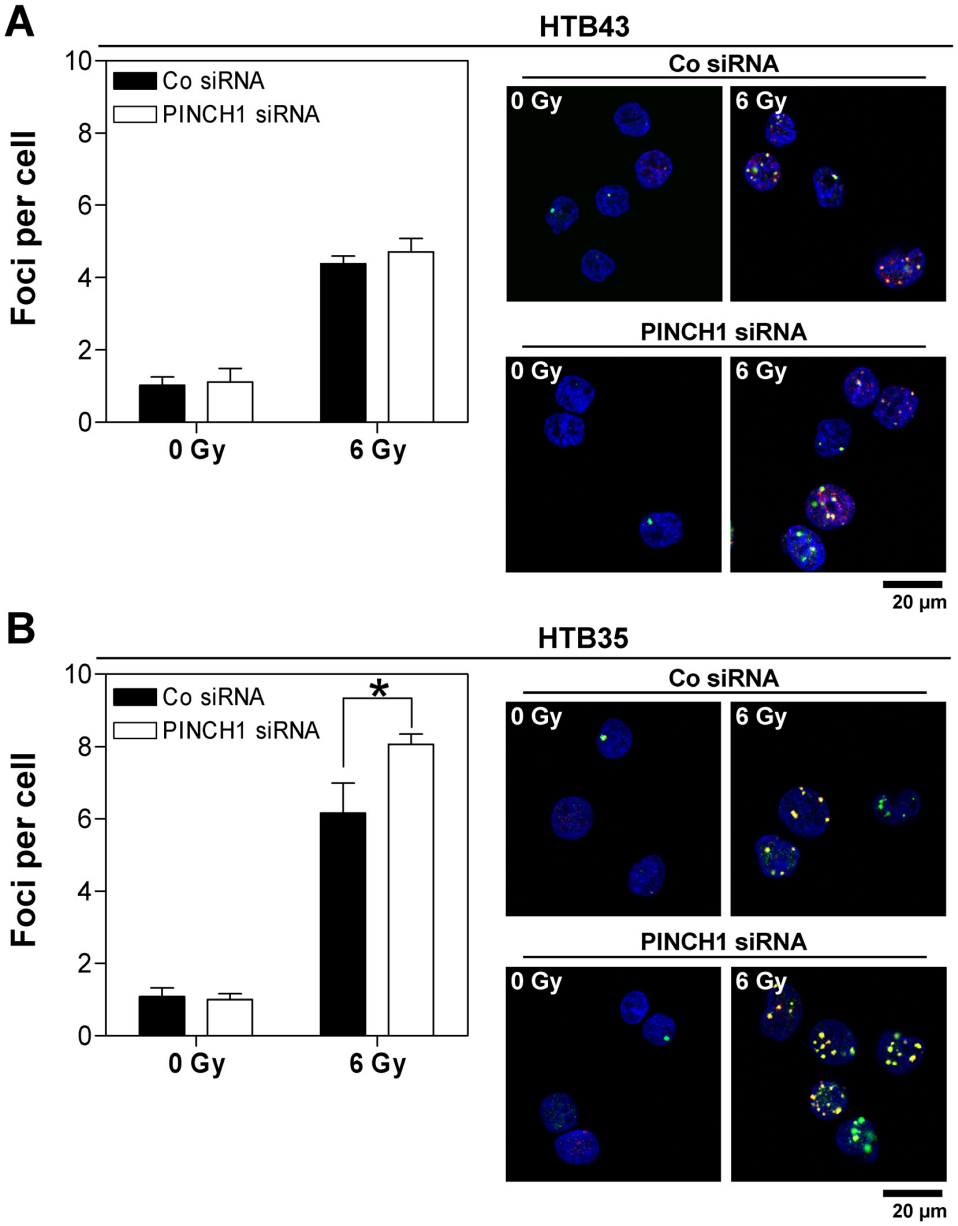
PINCH1 knockdown affects DNA double strand break repair cell line-dependently. Under PINCH1 depletion alone and in combination with X-ray irradiation, foci representing residual DSBs ( = 24 h after irradiation) were evaluated in (A) HTB43 and (B) HTB35 cells (mean±SD; *n* = 3; * *P*<0.05; t-test). Immunofluorescence images show the overlay of γH2AX/53BP1 double staining. Red, γH2AX; Green, 53BP1; Blue, DAPI.

Next we asked whether a PINCH1 knockdown modifies cell adhesion, proliferation, apoptosis and repair of DNA double strand breaks (DSBs) and found that HTB43 and HTB35 cell adhesion stayed unaffected upon PINCH1 depletion compared to siRNA controls (Fig. 6A–F) and that the number of cells per colony (Fig. 7A and B) as well as colony size (Fig. 7C and D; panel *i* and *ii*) and morphology on a single cell basis (Fig. 7C and D; panel *iii*) is not influenced by PINCH1 depletion. Moreover, PINCH1 knockdown HTB43 and HTB35 cell cultures demonstrated no increased level of apoptosis upon irradiation (Fig. 8A and B). Inconsistent between tested cell lines, we found a significantly (*P*<0.05) raised number of γH2AX/53BP1-positive foci per cell ( = residual DSBs at 24 h after irradiation) in PINCH1 depleted, 6-Gy irradiated HTB35 cells relative to irradiated controls (Fig. 9A and B). Thus, although the enhancement of radiosensitivity through PINCH1 gene knockout or gene silencing is consistent among species, the presented endpoint analyses were unable to provide a prominent mechanism of action.

### PINCH1 depletion differentially modifies protein phosphorylation under adhesion and suspension conditions

**Figure 10:**
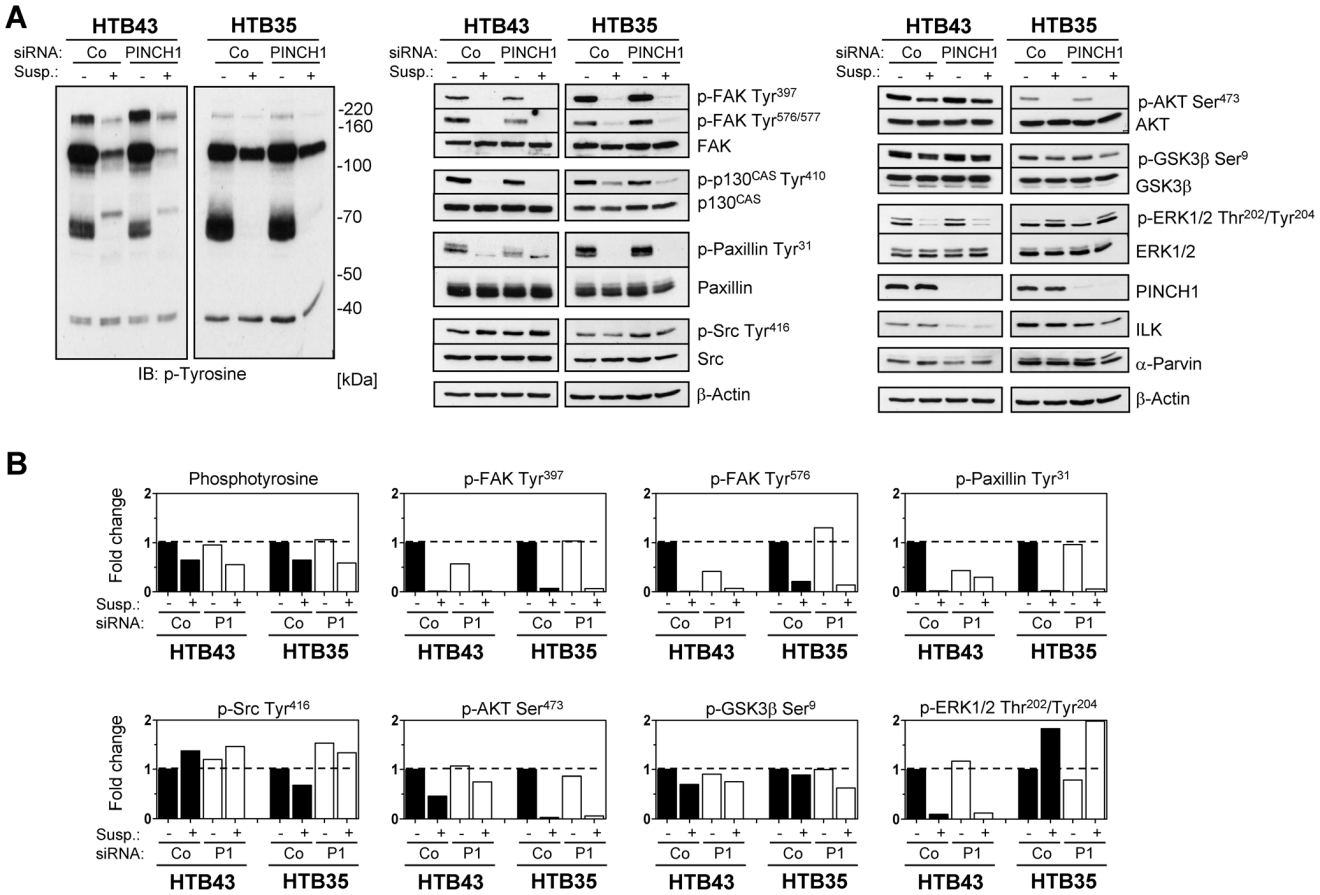
Signal transduction modification in adherent and suspension tumor cell lines after PINCH1 knockdown. (A) Western blot on total cell lysates from PINCH1 depleted HTB43 and HTB35 cells grown under adhesion or in suspension. (B) Densitometric analysis from protein bands shown in ‘A’ after normalization to total protein or β-Actin expression and subsequently to adhesion conditions of siRNA control cells ( = 1). Co, control; P1, PINCH1.

The examination of signaling molecules associated with integrin and growth factor receptors, as done for MEF analysis, followed. In concordance with MEF data sets, phosphotyrosine levels declined upon PINCH1 knockdown without dependence on adhesion or suspension (Fig. 10A and B). FAK Tyr^397^ and Tyr^576/577^, Paxillin Tyr^31^, AKT Ser^473^, and ERK1/2 Thr^202^/Tyr^204^ phosphorylation showed a complete or pronounced reduction in HTB43 and HTB35, respectively, when grown in suspension (Fig. 10A and B). This effect indicated no PINCH1 dependency. As compared to the findings in MEFs, an induced Src Tyr^416^ phosphorylation in control and PINCH1 knockdown cultures was observable in HTB43 cells under non-adherent conditions (Fig. 10A and B). Total protein expression changes could only be detected for ILK and α-Parvin upon PINCH1 depletion in an adhesion-independent manner (Fig. 10A). These findings show great similarity in the signaling of immortalized normal mouse cells and human tumor cells under adhesion versus suspension conditions on a PINCH1 knockout or knockdown background.

## Discussion

Understanding the molecular circuitry of the radiation survival response might strongly assist optimization of tumor therapy, particularly radiotherapy, and issues related to radioprotection. Owing to a great lack of knowledge in this area of research, we examined the radiation survival response of cells under adhesion versus suspension conditions in this study. In recent years, observations from our group and others pinpointed the importance of FA signaling for the survival of cells exposed to X-rays and chemotherapeutics. The multiprotein complex characteristic of FA suggests more molecules inevitable involved in such stress reactions than integrins and growth factor receptors. Concluding from previous findings that PINCH1 confers radioresistance [23], the present study elucidated whether PINCH1 also mediates its prosurvival effects under suspension conditions in different mouse and human cell lines. Here, we found that the enhanced cellular radiosensitivity mediated by PINCH1 depletion is independent from adhesion and can also be observed under suspension conditions. Despite a reduced DSB repair ability in one of the two tested tumor cell lines, other possible determinants of clonogenic data analysis such as proliferation as well as adhesion and apoptosis were taken into account or could be excluded, respectively. Our findings can also not be explained by modifications of adhesion and growth factor associated signaling involving FAK, Paxillin, p130^CAS^, AKT1 and MAPK under suspension and serum withdrawal relative to adhesion plus serum conditions. The presented data suggest that the adapter protein PINCH1 critically participates in the regulation of the cellular radiosensitivity of normal and malignant cells similarly under adherent and suspension conditions.

In various human normal and tumor cell line models, adhesion to ECM confers resistance against ionizing radiation (X-rays) and cytotoxic agents [1], [6], [8], [44], [45], [46], [47]. These phenomena, called cell adhesion mediated radioresistance (CAM-RR) and cell adhesion mediated drug resistance (CAM-DR), arise from interactions of cells with components of their microenvironment through binding of integrins to ECM or through cytokine binding to their cognate receptors with subsequent channeling of prosurvival biochemical cues [3], [10], [48]. However, the tested immortalized *PINCH1* ^fl/fl^ and *PINCH1* ^−/−^ MEF showed insensitivity to ECM adhesion with regard to radiation survival. With remaining PINCH1-dependent differences in radiosensitivity, these cells similarly survived under suspension as compared to adhesion conditions after exposure to increasing single doses of X-rays, a finding confirmed by PINCH1 reconstitution in *PINCH1* ^−/−^ MEF. From our analysis on the influence of growth factors/FCS on radiation survival, *PINCH1* ^fl/fl^ MEF seem to become more sensitive to serum withdrawal over time as compared to *PINCH1* ^−/−^ MEF, whose radiation survival remained unchanged.

The comparison of protein expression and phosphorylation in this MEF system suggests a PINCH1-related modification of protein tyrosine phosphorylation rather than protein expression. From further analysis, we identified moderate to strong dephosphorylation of FAK and the associated proteins Paxillin and p130^CAS^ as well as AKT, GSK3β and ERK1/2. Despite these clear differences resulting from PINCH1 depletion, which have been partly pursued as recently published [23] and provide a molecular basis for untangling the radiosensitization by PINCH1 knockout, the unsuspected similarity between radiation survival of adhesion and suspension cultures cannot be explained and requires further evaluation. Certainly, as this is only a snapshot analysis of a very limited number of signaling molecules, additional signal transduction events and changes in other important cell processes such as gene expression and post-translational protein modification have also to be taken into consideration.

As next step, we decided to examine the generality of this phenomenon in human cancer cell lines from different origin, i.e. head and neck and cervix. Subsequent to observing the heterogenic expression of the complex proteins PINCH1, ILK and α-Parvin among human cancer cell lines in contrast to a well known and clear pattern in both PINCH1 MEF and ILK mouse kidney fibroblasts [11], [12], [14], [16], [17], we performed a siRNA-mediated knockdown of PINCH1 in HTB43 and HTB35 cells. The resulting radiosensitization in these cell lines confirmed our previously reported data in other cell line models [23]. Intriguingly, it is shown that the absence/strong downregulation of PINCH1 does not result in any adhesion defects, which might account for the differences in clonogenic survival, an observation also supported by the presented similarity in plating efficiencies of PINCH1 knockdown HTB43 and HTB35 cell cultures relative to siRNA controls. Moreover, in line with unaltered cell adhesion, changes in cell morphology under reduced PINCH1 expression were undetectable.

The role of PINCH1 on tumor cell apoptosis has been recently documented by Chen and colleagues [49]. They showed a regulation of Bim translocation to mitochondria by PINCH1 in a HT-1080 fibrosarcoma cell model that seems relevant for antiapoptotic effects in tumor cells. In our hands, a PINCH1 knockdown failed to induce apoptosis in the two tumor cell lines used. A second highly relevant parameter in the context of radiobiology is the repair of DSBs as most life-threatening DNA lesions upon exposure to ionizing radiation [50]. With the presented inconsistencies in these two human tumor cell lines, it is impossible to assess the impact of PINCH1 on DSB repair. With one report on PINCH1's nuclear import and export sequence and its nuclear localization [51], PINCH1 might influence such kind of processes via its function as adapter protein and/or via its putative function as transcription factor.

Of further importance for the presented study is the fact that the degree of radiosensitization was maintained when cells were irradiated in suspension. However, the dose-effect curves of adherent PINCH1 knockdown cultures and suspension siRNA controls were superimposable, thus, indicating that the tested human tumor cell lines receive prosurvival/radioresistance signals through ECM adhesion, which are cut-off under detachment conditions.

In comparison with the analysis of MEFs, HTB43 but not HTB35 cells showed alterations of protein phosphorylations upon PINCH1 silencing. In addition to ILK and α-Parvin repression, PINCH1 depleted HTB43 cells revealed reduced levels of FAK Tyr^397^ and Tyr^576/577^ and Paxillin Tyr^31^. Under suspension, however, broad dephosphorylation occurred in both HTB43 and HTB35 cells with the exception of Src, whose Tyr^416^ phosphorylation was induced. Similar to MEFs, the analysis of signaling molecules involved in adhesion and growth factor receptor signaling provided no definite and molecular explanation for the radiosensitization through PINCH1 knockdown.

In conclusion, our data generated in MEF and human tumor cells suggest that the adapter protein PINCH1 critically participates in the regulation of the cellular radiosensitivity of normal and malignant cells independent from cell adhesion. For clarification of a function of PINCH1 aside from FAs and whether other adapter proteins are key to the cellular radiation response remains to be solved in future examinations.
